# Hinchey III Diverticulitis in a 31-Year-Old Patient With Williams Syndrome: A Case Report

**DOI:** 10.7759/cureus.63898

**Published:** 2024-07-05

**Authors:** Savvas C Tsaramanidis, Ioannis Gkoutziotis, Georgios Zacharioudakis, Ariadni Fouza, Konstantinos Mpallas

**Affiliations:** 1 5th Surgical Department, Hippocrates General Hospital/Aristotle University of Thessaloniki, Thessaloniki, GRC

**Keywords:** treatment choices, primary suturing, complicated diverticulitis, laparoscopic lavage, williams–beuren syndrome

## Abstract

Williams syndrome was first reported by Williams and Beuren in 1961-1962. It is a genetic disorder that is caused by a sporadic microdeletion of chromosome 7, which includes the elastin gene. The development of gastrointestinal pathology, such as diverticular disease, is associated with the deletion of this specific gene. Almost one-third of patients with Williams syndrome develop diverticular disease. The first episode of diverticulitis appears in 8% of patients, diagnosed with Williams syndrome, before the age of 40. According to the literature, in the case of complicated diverticulitis (Hinchey III) in patients with WS, the treatment is mainly surgical resection of sigmoid and colostomy (Hartmann procedure) or anastomosis. We present an interesting case with a 31-year-old male, with Williams syndrome and Hinchey III diverticulitis, who underwent laparoscopic lavage and primary closure of the perforation. To our knowledge, this is the first case in literature that a patient with Williams syndrome and complicated diverticulitis (Hinchey III) was treated this way and the results until now are encouraging.

## Introduction

Williams syndrome (WS) was first reported by Williams and Beuren in 1961-1962. It is a genetic disorder that is caused by a sporadic microdeletion of chromosome 7, which includes the elastin gene [1,2]. It is now recognized to be a multi-system disorder with particularly prominent cardiovascular, endocrine, and neurological manifestations [3]. The elastin gene, through the expression of tropoelastin, preserves connective tissue construction and cohesion [1]. The development of gastrointestinal pathology, such as diverticular disease, is associated with the deletion of this specific gene [1]. Almost one-third of patients with Williams syndrome develop diverticular disease [1]. It has been hypothesized that patients with WS may be at increased risk of diverticulitis [4]. WS has a reported incidence of one in 7500 live births [5]. The first episode of diverticulitis appears in 8% of patients, diagnosed with Williams syndrome, before the age of 40, in contrast to 2% in the general population of the same age [1].

## Case presentation

A 31-year-old male with WS and some characteristics of the syndrome, (mild supravalvular aortic stenosis, hypothyroidism, raised blood pressure, and intellectual disability), was presented to the emergency department (ED) complaining of abdominal pain for the last two days, mainly in the left iliac fossa, accompanied by diarrhea. He also complained of decreased appetite and a fever of up to 38.8 C. 

**Figure 1:**
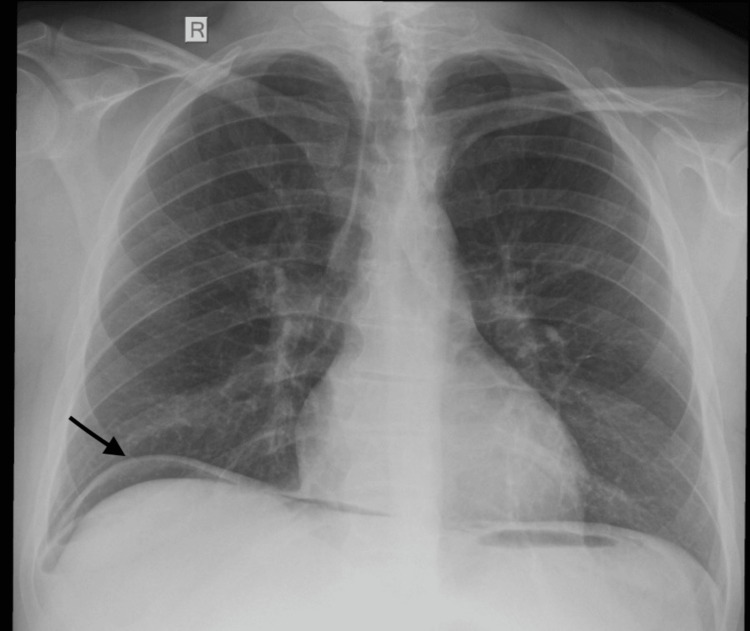
Free gas in x-ray (arrow), indicating perforation

Clinical examination revealed tenderness to palpation throughout the abdomen and rebound tenderness on the left iliac fossa. Laboratory blood tests developed elevated white blood cell count (26.20 x 10^3^/μL) and high c-reactive protein (CRP) 183.3mg/L (<6). Chest x-ray showed free gas under the diaphragm (Figure 1), indicating perforation.

**Figure 2:**
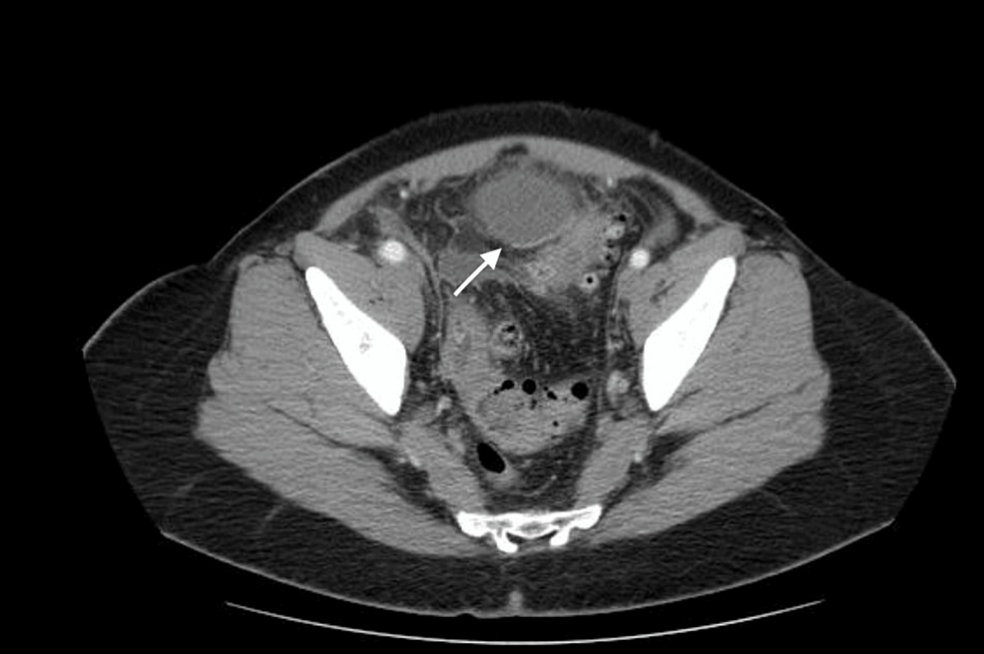
Acute perforated diverticulitis (Hinchey III)- purulent collection (arrow)

**Figure 3:**
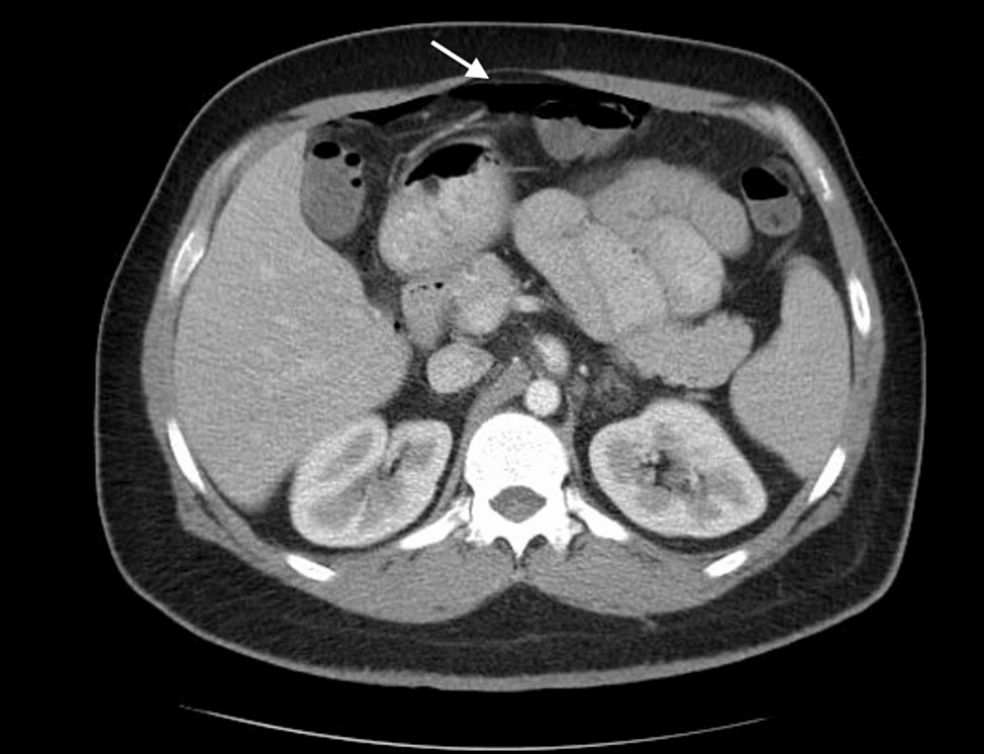
CT scan reveals free air in the abdomen (arrow)

Furthermore, a contrast CT scan was performed, which revealed free gas intraperitoneally and the location of the rupture. Multiple diverticula in the descending and sigmoid colon with perisigmoid fluid collection (6.4x3.9x5.2 cm) were observed, confirming the diagnosis of peritonitis due to ruptured diverticula (Figures 2, 3). 

**Figure 4:**
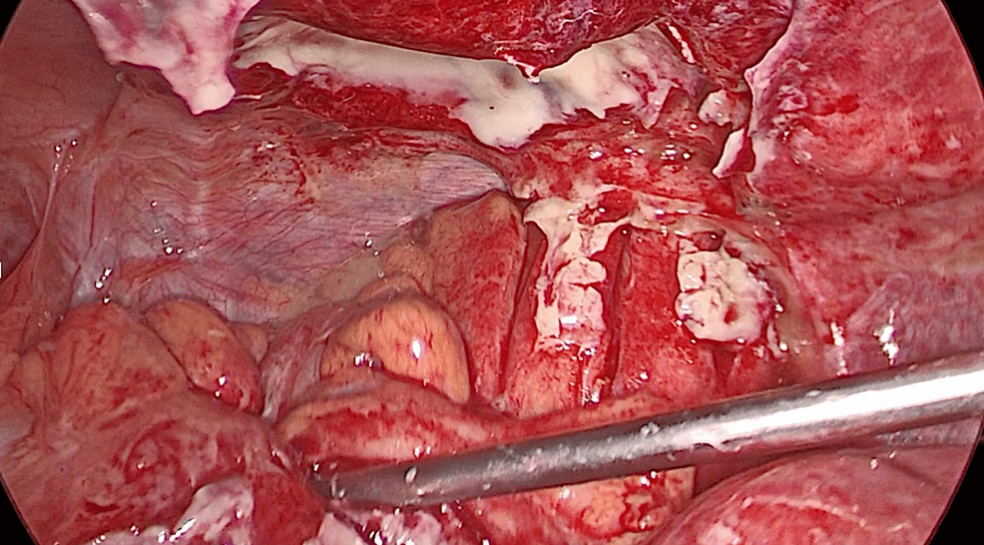
Laparoscopic lavage in purulent peritonitis (Hinchey III diverticulitis)

**Figure 5:**
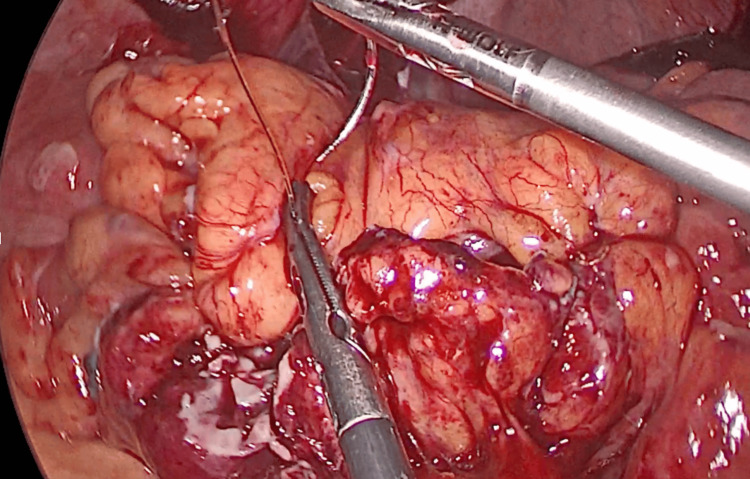
Primary closure with continuous suture

The patient was transferred to the operation theatre. Laparoscopy confirmed the sigmoid perforation, with purulent fluid in the peritoneal cavity (Hinchey classification stage III). Laparoscopic lavage and primary closure of the perforation with a continuous suture were performed (Figures 4, 5).

A drain was placed intraperitoneally, approximating the rupture.

The postoperative course of the patient was uneventful. Ηe was discharged fully independent on the 4th postoperative day (POD). The drain was removed in the 15th POD in the outpatient clinic, at the same time as the removal of stitches. He reports no further symptoms one year after surgery.

## Discussion

Diverticular disease offends approximately 50% of older adults, older than 60 years old. Nevertheless, about 5% of people younger than 40 years old have been reported to have this disease [5]. It is probably caused by increased pressure in the colon and by changes in elastin and collagen of the colonic wall, which have occurred over the years [2]. Some of the well-known risk factors contributing to the development of diverticular disease are constipation, physical inactivity, and a low-fiber, high-fat diet [6]. Many children with connective tissue abnormalities, such as Marfan and Ehlers-Danlos syndrome, and with genetic diseases such as cystic fibrosis, manifest diverticulum, probably because of the weakened colonic wall or the raised intraluminal pressure [5]. Among the others, the risk for colonic diverticulosis is increased in patients with WS [5]. According to a study by Parsch et al. [4], it is found that in patients with WS, diverticular disease occurs three to four times more often than in the general population [7].

Even though the cause of the high incidence of diverticulosis in patients with WS remains unknown, many studies suggest that a defect of the elastin protein is responsible for this disease. The role of elastin protein is to give elasticity to connective tissue structures. Fifteen genes deletion leads to WS, one of these genes is the elastin gene, which encodes the elastin protein. Around about 98% of individuals suffering from WS, demonstrate deletion of elastin gene. Lack of elastin protein results in reduced bowel wall elasticity, which is normally present and helps withstand the intraluminal colonic pressure in a healthy bowel. Consequently, WS patients develop diverticulosis due to the lack of this gene [5]. 

A more aggressive course of diverticulitis, with more complications, is observed in young patients [8]. Additionally, patients with Williams syndrome develop usually a complicated type of diverticulitis, so conservative treatment is not recommended [1]. According to the literature, in the case of complicated diverticulitis (Hinchey III) in patients with WS, the procedure more commonly performed, is surgical resection of sigmoid and colostomy (Hartmann procedure) or anastomosis [9]. 

We present a case in which we performed neither Hartmann procedure nor resection with anastomosis. Instead, we performed laparoscopic lavage and primary closure of the leak, strengthening it with an epiploic appendage patch over it. A surgical drain was also placed.

Laparoscopic lavage is an alternative approach to the treatment of Hinchey III diverticulitis [9]. As the Dutch acute diverticulitis guidelines suggest “laparoscopic lavage and drainage is a treatment modality which can be considered as an alternative to resection in these patients" [10]. However, cases with Hinchey III diverticulitis that were treated using laparoscopic lavage, drainage, and closure of the colonic perforation are reported in the literature. The outcomes were favorable [9].

To our knowledge, this is the first case in literature that a patient with Williams syndrome and complicated diverticulitis (Hinchey III) was treated in this way successfully.

## Conclusions

Diverticulitis is a disease that healthcare providers should consider in young patients with WS as a possible cause of abdominal pain. Constipation symptoms in these patients should be managed to prevent the development of diverticulosis and consequently the development of diverticulitis. Laparoscopic lavage, direct suturing of the perforation, and a drain placement could be a promising option, less aggressive than resection, in WS patients with Hinchey III diverticulitis.

## References

[1] Raber MM, Bowling SM, Dorn M (2022). Complicated diverticulitis in a 35-year-old patient with Williams syndrome: a case report. Cureus.

[2] Yamada H, Ishihara S, Akahane T (2011). Two cases of diverticulitis in patients with Williams syndrome. Int Surg.

[3] Pober BR, Morris CA (2007). Diagnosis and management of medical problems in adults with Williams-Beuren syndrome. Am J Med Genet C Semin Med Genet.

[4] Partsch CJ, Siebert R, Caliebe A, Gosch A, Wessel A, Pankau R (2005). Sigmoid diverticulitis in patients with Williams-Beuren syndrome: relatively high prevalence and high complication rate in young adults with the syndrome. Am J Med Genet A.

[5] Garcia MA, Kling KM, Newbury RO, Huang JS (2009). Complicated diverticular disease in a child with williams syndrome. J Pediatr Gastroenterol Nutr.

[6] Stagi S, Lapi E, Chiarelli F, de Martino M (2010). Incidence of diverticular disease and complicated diverticular disease in young patients with Williams syndrome. Pediatr Surg Int.

[7] Ignacio RC Jr, Klapheke WP, Stephen T, Bond S (2012). Diverticulitis in a child with Williams syndrome: a case report and review of the literature. J Pediatr Surg.

[8] Deshpande AV, Oliver M, Yin M, Goh TH, Hutson JM (2005). Severe colonic diverticulitis in an adolescent with Williams syndrome. J Paediatr Child Health.

[9] Sijberden J, Snijders H, van Aalten S (2021). Laparoscopic lavage in complicated diverticulitis with colonic perforation, always be closing?. Case Rep Gastroenterol.

[10] Dutch Federation of Medical Specialists (2020). Acute diverticulitis. https://richtlijnendatabase.nl/richtlijn/acute_diverticulitis/startpagina_-_acute_diverticulitis.html.

